# Quantitative Proteomics Reveals that the OGT Interactome Is Remodeled in Response to Oxidative Stress

**DOI:** 10.1016/j.mcpro.2021.100069

**Published:** 2021-03-12

**Authors:** Marissa Martinez, Santosh Renuse, Simion Kreimer, Robert O’Meally, Peter Natov, Anil K. Madugundu, Raja Sekhar Nirujogi, Raiha Tahir, Robert Cole, Akhilesh Pandey, Natasha E. Zachara

**Affiliations:** 1 Department of Biological Chemistry, Johns Hopkins University School of Medicine, Baltimore, Maryland, United States; 2 Currently at Foghorn Therapeutics, Cambridge, Massachusetts, United States; 3 McKusick-Nathans Institute of Genetic Medicine, Johns Hopkins University School of Medicine, Baltimore, Maryland, United States; 4 Currently at the Department of Laboratory Medicine and Pathology, Mayo Clinic, Rochester, Minnesota, United States; 5 Currently at the Center for Individualized Medicine, Mayo Clinic, Rochester, Minnesota, United States; 6 The Mass Spectrometry and Proteomics Facility, The Johns Hopkins University School of Medicine, Baltimore, Maryland, USA; 7 Currently at the Advanced Clinical Biosystems Institute, Smidt Heart institute, Cedars Sinai Medical Center, Los Angeles, California, USA; 8 Currently at the Department of Internal Medicine, Yale New Haven Hospital, Yale School of Medicine, New Haven, Connecticut, USA; 9 Currently at the Medical Research Council (MRC) Protein Phosphorylation and Ubiquitylation Unit, School of Life Sciences, University of Dundee, Dundee, UK; 10 Biochemistry, Cellular and Molecular Biology Graduate Program, Johns Hopkins University School of Medicine, Baltimore, Maryland, United States; 11 Currently at Ginkgo Bioworks, Massachusetts, United States; 12 Manipal Academy of Higher Education, Manipal, Karnataka, India; 13 Department of Oncology, Johns Hopkins University School of Medicine, Baltimore, Maryland, United States

**Keywords:** Glycosylation, Cytoprotection, Network, Quantitative proteomics, Oxidative stress, 4MU, 4-methylumbelliferyl, Bag6, large proline-rich protein BAG6, CARM1, coactivator-associated arginine methyltransferase 1, CKII, casein kinase II, eIF3b, eukaryotic translation initiation factor 3 subunit B, FL, full length, FOXO, daf-16, forkhead box protein O, GFAT, glutamine-fructose-6-phosphate transaminase, H_2_O_2_, hydrogen peroxide, HCF-1, host cell factor 1, HDAC1, histone deacetylase 1, HSF1, heat shock factor protein 1, MEFs, mouse embryonic fibroblasts, Null, OGT KO, OGA, O-GlcNAcase, O-GlcNAc, O-linked N-acetyl-β-D-glucosamine, OGT, O-GlcNAc transferase, PRM, parallel reaction monitoring, SETD1A, histone-lysine N-methyltransferase SETD1A, SILAC, stable isotopic labeling of amino acids in cell culture, TBST, Tris-buffered saline with Tween-20, TCL, total cell lysis, TEABC, triethylammonium bicarbonate, TNKS1BP1, 182-kDa tankyrase-1–binding protein, TPR, tetratricopeptide repeats, UAP1, UDP-N-acetylhexosamine pyrophosphorylase

## Abstract

The dynamic modification of specific serine and threonine residues of intracellular proteins by O-linked N-acetyl-β-D-glucosamine (O-GlcNAc) mitigates injury and promotes cytoprotection in a variety of stress models. The O-GlcNAc transferase (OGT) and the O-GlcNAcase are the sole enzymes that add and remove O-GlcNAc, respectively, from thousands of substrates. It remains unclear how just two enzymes can be specifically controlled to affect glycosylation of target proteins and signaling pathways both basally and in response to stress. Several lines of evidence suggest that protein interactors regulate these responses by affecting OGT and O-GlcNAcase activity, localization, and substrate specificity. To provide insight into the mechanisms by which OGT function is controlled, we have used quantitative proteomics to define OGT’s basal and stress-induced interactomes. OGT and its interaction partners were immunoprecipitated from OGT WT, null, and hydrogen peroxide–treated cell lysates that had been isotopically labeled with light, medium, and heavy lysine and arginine (stable isotopic labeling of amino acids in cell culture). In total, more than 130 proteins were found to interact with OGT, many of which change their association upon hydrogen peroxide stress. These proteins include the major OGT cleavage and glycosylation substrate, host cell factor 1, which demonstrated a time-dependent dissociation after stress. To validate less well-characterized interactors, such as glyceraldehyde 3-phosphate dehydrogenase and histone deacetylase 1, we turned to parallel reaction monitoring, which recapitulated our discovery-based stable isotopic labeling of amino acids in cell culture approach. Although the majority of proteins identified are novel OGT interactors, 64% of them are previously characterized glycosylation targets that contain varied domain architecture and function. Together these data demonstrate that OGT interacts with unique and specific interactors in a stress-responsive manner.

## Highlights

• O-GlcNAc levels change dynamically in response to injury.
• Injury does not induce changes in activity of the enzymes that cycle O-GlcNAc.
• Quantitative proteomics identified and validated interactors of OGT.
• The interactome of OGT changes significantly in response to oxidative stress.

## Graphical Abstract

**Figure:**
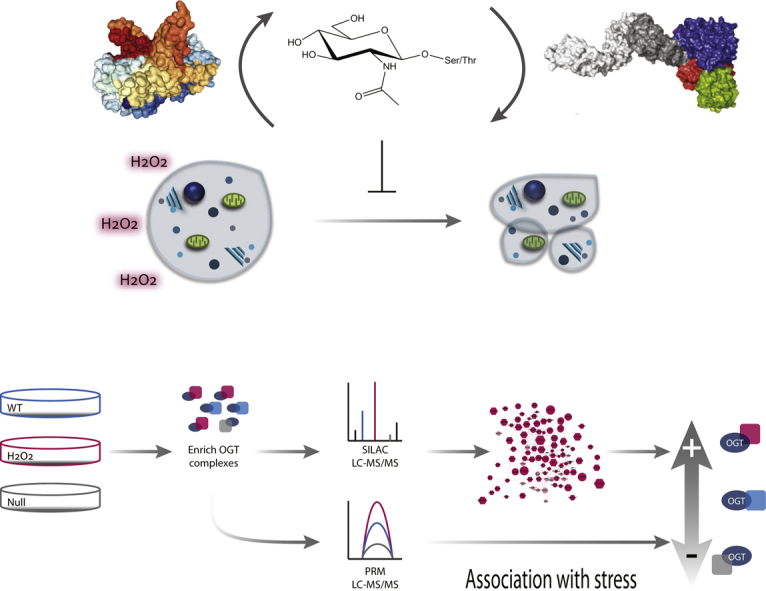

## In Brief

The goal of these studies was to provide insight into the regulation of the O-GlcNAc transferase (OGT) basally and in response to oxidative stress, as well as the role that O-GlcNAc plays in promoting cytoprotection. Using quantitative proteomics, the basal and injury-induced interactome of OGT has been defined and validated. Protein interactors are anticipated to regulate either the activity or substrate targeting of OGT, or to be substrates of OGT, thus affecting cytoprotection.

The modification of intracellular proteins by monosaccharides of O-linked N-acetyl-β-D-glucosamine (O-GlcNAc) has emerged as a novel post-translational modification involved in numerous stress and disease models (1, 2). Induction of cell or tissue injury, such as oxidative stress, heat shock, ischemia reperfusion injury, and trauma hemorrhage, elevates O-GlcNAcylation on numerous proteins (3, 4, 5). Endogenous pathways that elevate O-GlcNAc, such as ischemic preconditioning, and exogenous induction of O-GlcNAcylation by altering O-GlcNAc cycling, protect cells during stress and serve to promote cell survival (4, 6, 7, 8). Although many of the mechanisms by which O-GlcNAc regulates the protein function have been elucidated, the regulation of O-GlcNAc cycling and how it promotes cytoprotective responses during stress remain less clear.

O-GlcNAc is added and removed to specific serine and threonine residues by just two enzymes, the O-GlcNAc transferase (OGT) and the O-GlcNAcase (OGA), respectively (9, 10). There are four characterized isoforms of OGT (9, 11, 12, 13, 14) and two of OGA (10, 15). With no consensus sequence defined for O-GlcNAcylation, it remains unclear how these enzymes and their isoforms can target thousands of substrates with diverse functions and subcellular localizations (cytoplasm, nucleus, mitochondria, etc.) (1, 2). Using quantitative proteomics, we have shown that OGA exists in varied multiprotein complexes (16). These studies demonstrated that a subset of OGA associates with fatty acid synthase in response to oxidative stress, functioning to diminish OGA catalytic activity (16). Similarly, data suggest that OGT also exists in multiprotein complexes and is regulated by its protein interactors. Indeed, both ad hoc and high-throughput approaches have identified OGT interactions that regulate either OGT or the interacting protein. OGT interacts with p38 mitogen-activated protein kinase during nutrient deprivation, affecting OGT substrate targeting (17). Work utilizing a yeast-two-hybrid screen described several interactors, including myosin phosphatase target subunit 1, which is both an OGT substrate and regulatory partner (18). Proteomics analyses after overexpression of OGT identified both host cell factor 1 (HCF1) and peroxisome proliferator–activated receptor gamma coactivator 1-alpha as interacting partners. Subsequent studies demonstrated that OGT and HCF1 binding synergistically elevates proliferator–activated receptor gamma coactivator 1-alpha activity (19). However, the effects of many protein interactions on OGT function and how they change upon a stress stimulus remain largely unknown.

To provide insight into the regulation of OGT during injury, we have mapped the basal and oxidative stress–induced interactomes of OGT via endogenous enrichment coupled with orthogonal quantitative proteomics strategies for identification (stable isotopic labeling of amino acids in cell culture [SILAC]) and validation (parallel reaction monitoring [PRM]). Both approaches utilized mouse embryonic fibroblasts (MEFs), in which OGT can be inducibly deleted, and the stress response has been comprehensively studied (3, 20, 21). We identified well-characterized interactors of OGT, including HCF1, trafficking kinesin-binding proteins 1 and 2, tet methylcytosine dioxygenase 3, as well as novel interactors, such as short-chain–specific acyl-CoA dehydrogenase, 182-kDa tankyrase-1–binding protein (TNKS1BP1), and phosphoenolpyruvate carboxykinase 2. Coupled with network analysis, these data suggest that varied and specific protein partners regulate the function of OGT in response to stress.

## Experimental Procedures

### Antibodies and Reagents

Unless otherwise stated, all reagents used were from either MilliporeSigma (St Louis, MO) or Thermo Fisher Scientific (Waltham, MA) and were of molecular biology grade or higher. The following antibodies were used for Western blot analysis: OGT (O6264) and actin (ACTA1; A5060; MilliporeSigma), large proline-rich protein BAG6 (Bag6; A302-039A), coactivator-associated arginine methyltransferase 1 (CARM1) (A300-421A) and HCF1 (A301-400A; Bethyl, Montgomery, TX), heat shock cognate 71 (sc-7298), and TNKS1BP1 (sc-514517; Santa Cruz, Dallas, TX). O-GlcNAc antibody, CTD 110.6, was antigen affinity–purified by The Johns Hopkins University School of Medicine O-GlcNAc Core (Core C4). OGT antibody (AL24; gifted by Dr Gerald Hart (9)) was antigen affinity–purified and used for immunoprecipitation of OGT–protein complexes. Nonspecific rabbit IgG was used as an isotype control for immunoprecipitation experiments (2729S; Cell Signaling Technology, Danvers, MA).

### Cell Culture and Oxidative Stress Treatments

MEFs were maintained in low-glucose DMEM (1 g/L; Corning, Tewksbury, MA) with fetal bovine serum (10% v/v) and penicillin/streptomycin (1% v/v; Corning). As previously reported, for SILAC, cells were passaged ≥8 times in low-glucose media supplemented with light (L), medium (M), and heavy (H) isotopes of arginine (L: [^12^C_6_, ^14^N_4_], M: [^13^C_6_], H: [^13^C_6_, ^15^N_4_]) and lysine (L: [^12^C_6_, ^14^N_2_], M: [D_4_], H: [^13^C_6_, ^15^N_2_]) (Cambridge Isotopes, Tewksbury, MA) (22). SILAC label swapping across replicates has been demonstrated to reduce experimental errors due to incomplete labeling, arginine-to-proline conversion, and contaminants due to cell culture media (23). As such, we chose to switch the isotopic labels between WT, OGT knockout (null), and hydrogen peroxide (H_2_O_2_) stress conditions for each biological replicate. OGT was inducibly deleted as described previously (20). MEFs were treated with 2.5-mM H_2_O_2_ in complete media for the indicated length of time or for 1.5 h in SILAC and PRM studies at 40-h postinduction of OGT deletion (22).

### Preparation of Cell Lysates

Frozen cell pellets were extracted in 50mM Tris HCL, 150-mM NaCl, pH 7.5 (Tris-buffered saline), with 1% (v/v) NP-40 and 2-mM EDTA (total cell lysis [TCL] buffer) supplemented with inhibitors (protease inhibitor cocktail sets II and III, 10-μM hexosaminidase inhibitor (376820; MilliporeSigma), 0.5-μM Thiamet G (synthesized by SD ChemMolecules LLC; (24)), 0.1-mM PMSF, 10-mM NaF, 10-mM β-glycerophosphate, and 3-μM trichostatin A1). Lysates were sonicated and cellular debris pelleted by centrifugation (18,000*g*, at 4 °C). The protein concentration was determined by using the Pierce 660 colorimetric protein assay, according to the manufacturer’s instructions (Thermo Fisher Scientific).

### OGT Activity Assays

Cells were lysed as described above without phosphatase inhibitors (NaF and β-glycerophosphate) and desalted by size-exclusion chromatography (Zeba, Waltham, MA) into 20-mM Tris-HCl, pH 7.8, 20% (v/v) glycerol. The cell lysate was incubated (1 h, at 25 °C) in triplicate with 0.5 Ci of [^3^H]UDP-GlcNAc (American Radiolabeled Chemicals, St Louis, MO), 2.5 units of calf intestinal alkaline phosphatase (New England BioLabs, Ipswich, MA), 0.250-mM 5’ AMP, and OGT assay buffer (100-mM sodium cacodylate, pH 6.4, 0.3% (w/v) BSA) with and without (negative control) 1-mM casein kinase II (CKII) (PGGSTPVSSANMM) or 1-mM α-crystallin (AIPVSREEK) acceptor peptides (The Johns Hopkins University School of Medicine Synthesis and Sequencing Facility). Recombinant His6-OGT was used as a positive control (25). Assays were quenched with 3 volumes of 50-mM formate, 1 M NaCl. Tritiated glycopeptide was separated from unincorporated UDP-GlcNAc by solid-phase extraction (Phenomenex, Torrance, CA) as reported previously (26). The incorporation of radiolabeled GlcNAc was assessed by liquid scintillation counting (Beckman Coulter, Indianapolis, IN). OGT assays were performed with n = 3 biological replicates and n = 3 technical replicates.

### OGA Activity Assays

Cell lysates desalted into 20-mM Tris HCl, pH 7.8, 20% (v/v) glycerol (as described above) were assessed for OGA activity in 50-mM sodium cacodylate, pH 6.4, 0.1 mg/ml BSA, and 100-mM GalNAc using fluorescent substrates (1-mM): 4-methylumbelliferyl (4-MU)–GlcNAc or 4-MU–GalNAc (MilliporeSigma) (16, 27). β-N-Acetylhexosaminidase (New England Biolabs, Ipswich, MA) was used as a positive control (5 units/well). Assays were quenched with 3 volumes of 200-mM glycine, pH 10.75, and the fluorescence intensity measured (excitation 360 nm and emission 460 nm) using the Synergy HT microplate reader (BioTek Instruments, Winooski, VT). OGA activity was normalized by subtracting the fluorescence signal resulting from lysosomal hexosaminidase contamination (4-MU–GalNAc) (16, 27). OGA assays were performed with n = 3 biological replicates and n = 2 technical replicates.

### Western Blot Analysis

SDS-PAGE was performed using tris-glycine and, for high molecular weight proteins, tris-acetate polyacrylamide midi-gels (Bio-Rad, Hercules, CA). Proteins were electroblotted to 0.45-μm nitrocellulose membranes (Bio-Rad). Before blocking, membranes were stained for total protein with SYPRO Ruby (Thermo Fisher), according to the manufacturer’s instructions. Subsequently, membranes were blocked in nonfat milk (3% w/v) in Tris-buffered saline with Tween-20 (TBST) (0.05% v/v; TBST) and subsequently incubated overnight with the primary antibody (4 °C). Membranes were washed with TBST and incubated with horse radish peroxidase–conjugated secondary antibody (1 h, at room temperature [RT]). Membranes were washed with TBST and developed using autoradiography film (Amersham, Little Chalfont, UK) or a chemiluminescent imaging system (Amersham Imager 600, RGB). Western blots were performed in n ≥ 3 biological replicates. All quantification was performed with ImageJ v 1.52 (imagej.nih.gov). Unless stated in the figure legend, protein signals were normalized to total protein (SYPRO Ruby). Of note, two bands were detected for TNKS1BP including full-length (FL) TNKS1BP at ∼250 kDa. While several commercial antibodies detect smaller species, we only quantified the FL TNKS1BP as the identity of the smaller species could not be confirmed.

### Immunoprecipitation of OGT and Its Interactors

Cell lysates were prepared as described above and precleared with the isotype control rabbit IgG antibody bound to protein G magnetic beads (2 h, at 4 °C). To enrich OGT and HCF1 to saturation, typically 2.4-μg AL24 and 6.7-μg αHCF1 antibody were used per mg of the cell lysate (16 h, at 4 °C). An equivalent amount of rabbit IgG antibody was used as an immunoprecipitation control. Before use, Protein G magnetic beads were blocked with 0.5% v/v Tween-20 (16 h, at 4 °C) and washed in TCL buffer. Magnetic beads were incubated with antibody–protein complexes (2 h, at 4 °C). Unbound proteins were collected, and the magnetic beads washed with TCL buffer. Bound OGT–protein complexes were eluted from the beads in 4X Laemmli sample buffer (5 min, 100^o^C). For SILAC samples, lysates were mixed at equal amounts of protein in a 1:1:1 ratio before the addition of the antibody. SILAC immunoprecipitations for LC-MS/MS analysis were performed in n = 4 biological replicates. All other immunoprecipitations were performed in n ≥ 3 biological replicates.

To reduce contamination from immunoglobulin, a subset of IPs (validation/PRM) were processed using the Pierce Classic immunoprecipitation kit (ThermoFisher Scientific). Lysates were precleared (1 h, at 4^o^C) using a resin conjugated to immunoglobulin (100 μl per mg of lysate) before OGT complexes were incubated with antigen-purified AL24 (2 μg/mg of lysate; overnight, 4^o^C). OGT:antibody complexes were then captured using protein-A/G sepharose (Pierce Classic immunoprecipitation kit). Complexes were washed using the proprietary cell lysis/wash buffer (5x) and subsequently eluted at pH 2.8 (15 min). Complexes were neutralized with Tris HCl (pH7.8) and separated by SDS-PAGE. A subset of the PRM samples (below; replicates 5–7) were processed in an analogous manner and concentrated using a methanol precipitation.

### Trypsin Digestion of Lysates

**Fig. 1:**
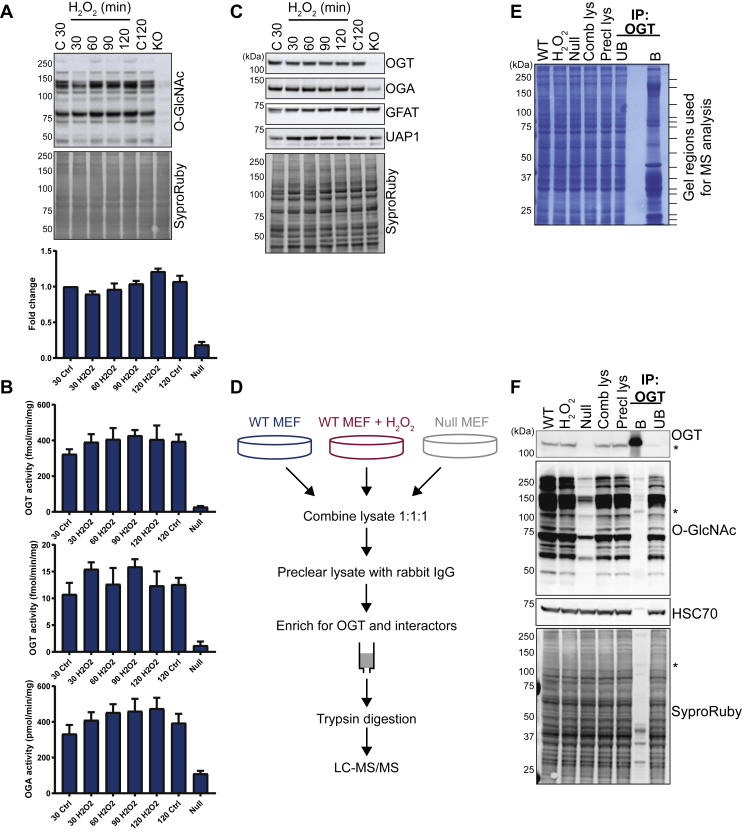
**The activity of OGT, but not the expression, changes with hydrogen peroxide stress.** MEFs were treated with 2.5-mM hydrogen peroxide (H_2_O_2_, 30–120 min). As a control, WT and null MEFs were treated with the complete media for 30 min and 120 min (C120). *A*, changes in O-GlcNAcylation in total cell lysate (20 μg) were determined by Western blot and quantified by ImageJ (one-way ANOVA, n = 3). The total protein was assessed by SYPRO Ruby. *B*, the activity of OGT in cell lysates (10 μg) was measured via ^3^H-UDP-GlcNAc transfer to either CKII (*top*) or α-crystallin (*middle*) peptides. OGA activity (*bottom*) in cell lysates (10 μg) was measured via the conversion of 4-MU-GlcNAc to 4-MU (one-way ANOVA, n = 3). *C*, the abundance of the enzymes that mediate O-GlcNAcylation- OGT, OGA, GFAT, and UAP1 were measured via Western blot. The total protein was assessed by SYPRO Ruby. Quantification via ImageJ is not shown as no significant differences were detected. *D*, the schematic representation of the enrichment strategy for identifying OGT interactors that change with H_2_O_2_ stress (n = 3). *E*, WT and null MEFs labeled with light, medium, or heavy isotopes of arginine and lysine. WT cells were treated with 2.5-mM H_2_O_2_ (1.5 h) and then lysed. Lysates were combined in equal amounts (Comb lys), precleared with isotype control rabbit IgG (Precl lys), and then enriched for OGT and protein interactors by immunoprecipitation (bound, *B*). Lysates from each step, as well as bound proteins, were separated by SDS-PAGE and stained with Coomassie. Subsequently, fractions of the lane were excised based on roughly equal staining intensities (*dash* marks). Proteins were digested in-gel with trypsin and peptides identified by LC-MS/MS. *F*, combined, precleared SILAC lysates were enriched for OGT and interactors and then immunoblotted for OGT and O-GlcNAc. Flow-through samples from the enrichment was also assessed (unbound, UB). HSC70 and SYPRO Ruby stained membranes function as loading controls for the corresponding blots (*A, C*, and *F*). The *asterisk* indicates migration of OGT. CKII, casein kinase II; GFAT, glutamine-fructose-6-phosphate transaminase; H_2_O_2_, hydrogen peroxide; HSC70, heat shock cognate 71; MEF, mouse embryonic fibroblast; OGA, O-GlcNAcase; OGT, O-GlcNAc transferase; O-GlcNAc, O-linked N-acetyl-β-D-glucosamine; SILAC, stable isotopic labeling of amino acids in cell culture; UAP1, UDP-N-acetylhexosamine pyrophosphorylase.

Bound OGT–protein complexes were further separated on large format tris-glycine gels (18 x 16 cm, GE Healthcare, Pittsburgh, PA) and stained with SimplyBlue SafeStain (Thermo Fisher Scientific) to visualize bands. Approximately equal sections of the gel (16 total) were excised and cut into 1-mm pieces (Fig. 1*E*). Gel pieces were completely destained in 50-mM triethylammonium bicarbonate (TEABC) with 40% (v/v) acetonitrile, shaking. Samples were reduced with 5-mM DTT in 50-mM TEABC (1 h, 37 °C) and alkylated with 20-mM iodoacetamide in 50-mM TEABC (20 min, at RT). Gel pieces were dehydrated with 100% acetonitrile and subsequently rehydrated in 10 ng/μl trypsin (MS Grade, #90057, ThermoFisher Scientific–Pierce; cleaves C-term Arg and Lys with high specificity) in 50-mM TEABC on ice (1 h). Excess trypsin was removed and replaced with 50-mM TEABC before overnight incubation (37 °C). Peptides were extracted from the gel with an increasing acetonitrile concentration until the gel pieces were fully dehydrated. Peptides were purified away from contaminants and salts by C_18_-reversed phase chromatography in STop and Go Extraction tips and dried down by SpeedVac (Thermo Fisher Scientific) and stored at −80 °C until LC-MS/MS analysis.

### LC-MS/MS Analysis

Peptides from in-gel–digested protein bands were analyzed on an Orbitrap Fusion Lumos ETD mass spectrometer (Thermo Scientific, Bremen, Germany) interfaced with Easy-nLC 1200 nanoflow liquid chromatography system (Thermo Scientific, Bremen, Germany). Peptides were loaded on a nanotrap column (PepMap C_18_, 5 μm, 100 °A, Thermo Scientific) using solvent A (0.1% v/v formic acid) with a flow rate 10 μl/min. Peptides were separated on an analytical column (PepMap C_18_ EasySpray, 2 μm, 75 μm x 50 cm, 100 °A, Thermo Scientific) with a 70-min gradient from 7 to 25% solvent B (99.9% v/v acetonitrile/0.1% v/v formic acid) with a flow rate of 300 nl/min. The spray voltage was set to 2.3 kV, whereas the ion-transfer tube temperature was set to 200 °C. The mass spectrometer was operated in data-dependent acquisition mode. A survey full-scan MS (m/z 350–1550) was acquired in the Orbitrap analyzer with resolution 120,000 at m/z 200. The automated gain control target for MS1 was set as 2 x 10^5^ with ion injection time as 50 ms. The most intense ions with the charge state ≥2 were isolated in 3-s cycle and fragmented using higher energy collisional dissociation fragmentation with 32% normalized collision energy and detected at a mass resolution of 30,000 at 200 m/z. The automated gain control target for MS/MS was set as 5 x 10^4^, and the ion-filling time set as 100-ms dynamic exclusion was set for 30 s with a ±7 ppm mass window. For all measurements with the Orbitrap detector, a lock-mass ion from ambient air (m/z 445.120025) was used for internal calibration as described (28).

### Data Analysis

To obtain protein identification and quantitation, all spectra were searched against the NCBI murine RefSeq database (v73; 99,957 entries) using the search algorithms SEQUEST and MASCOT incorporated into the Proteome Discoverer software package (v2.0, Thermo Fisher Scientific). The following search parameters were used: 1 missed cleavage; carboxamido methylation of cysteines (fixed); oxidation of methionine (variable); SILAC labels (variable): D_4_-lysine, ^13^C_6_,^15^N_2_-lysine, ^13^C_6_-arginine, ^13^C_6_,^15^N_4_-arginine; MS tolerance of  ±10 ppm; and MS/MS tolerance of ±0.1 Da. The false discovery rate was calculated using the Percolator (29) node in Proteome Discoverer. Briefly, the data were searched in a target-decoy approach and filtered by applying a 1% false discovery rate at the peptide and protein levels. Proteins were quantified as the median summed peptide spectral matches. The SILAC MS proteomics data have been deposited to the ProteomeXchange Consortium via the PRIDE (30) partner repository with the dataset identifier PXD013645.

SILAC ratios for all proteins were log_2_ transformed. To account for any loading biases in the SILAC mixes, protein log_2_ ratios were normalized to the median across all proteins for each replicate. Interactors were considered valid if the log_2_ WT or H_2_O_2_/null ratio ≥1 in 2 or more replicates. Interactors were considered to have changed with stress if log_2_ H_2_O_2_/null ≥1.5 (increased) or ≤ -0.67 (decreased). Values (Table 1) are presented as the median log_2_ ratios across 3 biological replicates.

**Table 1:** Select basal and stress-induced interactors of OGT

RefSeq	Gene symbol	Protein name	Median log_2_ H_2_O_2_/WT
XP_006541561.1	BCORL1	BCL-6 corepressor-like protein 1 isoform X1	5.31
NP_001074729.1	TNKS1BP1	182-kDa tankyrase-1–binding protein	3.41
XP_006537384.1	BAG6	Large proline-rich protein BAG6 isoform X8	3.36
XP_006518876.1	SEC24C	Protein transport protein Sec24C isoform X1	2.65
XP_011239611.1	WNK1	Serine/threonine-protein kinase WNK1 isoform X1	2.6
NP_659033.1	ACAT1	Acetyl-CoA acetyltransferase, mitochondrial precursor	1.61
NP_001035028.1	ASXL1	Putative polycomb group protein ASXL1	1.48
NP_780305.1	STBD1	Starch-binding domain-containing protein 1	1.47
NP_058061.2	PRKAG1	5'-AMP–activated protein kinase subunit gamma-1	1.0
XP_011238755.1	HIVEP3	Transcription factor HIVEP3 isoform X1	0.88
XP_006530933.1	CNOT1	CCR4-NOT transcription complex subunit 1 isoform X1	0.55
NP_081708.1	WDR77	Methylosome protein 50	0.39
XP_006509619.1	FKBP8	Peptidyl-prolyl *cis-trans* isomerase FKBP8 isoform X1	0.27
XP_006512287.1	TRAK1	Trafficking kinesin-binding protein 1 isoform X1	0.14
**XP_006527799.1**	**OGT**	**UDP-N-acetylglucosamine-peptide N-acetylglucosaminyltransferase**	−**0.08**
NP_032250.2	HCFC1	Host cell factor 1 precursor	−0.17
NP_821172.2	SETD1A	Histone-lysine N-methyltransferase SETD1A	−0.22
XP_006495966.1	KANSL3	KAT8 regulatory NSL complex subunit 3 isoform X4	−0.39
XP_006506530.1	CHCHD3	MICOS complex subunit Mic19 isoform X1	−0.60
NP_067506.2	CARM1	Histone-arginine methyltransferase CARM1 isoform 1	−0.66
NP_033921.3	ASPM	Abnormal spindle-like microcephaly-associated protein homolog	−1.59

H_2_O_2_, hydrogen peroxide; OGT, O-GlcNAc transferase.

The bait protein, the O-GlcNAc transferase, is highlighted in bold.

A subset of the 134 OGT interactors is listed above. Stress-induced changes in protein interactors were classified by a log_2_ H_2_O_2_/WT ratio ≥1.5 (increased) or ≤ -0.67 (decreased). In addition, proteins identified only in H_2_O_2_-treated or WT conditions are considered increased or decreased, respectively.

All network analysis was performed within the Cytoscape molecular interaction analysis platform (31). Interaction networks were generated with STRING database (32), where the node size is indicative of either WT or H_2_O_2_/null ratios within each network (Figs. 3 and 4, respectively), respectively. The network (organic layout) was generated using the yFiles algorithm application for undirected graphs (yWorks GmbH, Tübingen, Germany). Functional enrichment of processes performed by OGT interactors was analyzed via the STRING enrichment application. PROSITE and UniProtKB bioinformatics tools were used to identify the domains, motifs, and regions of similarity within proteins interacting basally and via stress with OGT. Proteins may contain more than one structural component and are thus listed for each component accordingly.

PRM was used to confirm the changes in coimmunoprecipitation of several identified OGT interactors under stress conditions (33). Initially (replicates 1–4), the immunoprecipitation of OGT was repeated as described above; however, OGT and interactors were eluted with 4% w/v SDS in 50-mM Tris HCl, pH 7.6. Samples were reduced with 5-mM DTT in 100-mM ammonium bicarbonate (ABC) (1 h, 56 °C) and alkylated with 10-mM iodoacetamide in 100-mM ABC (30 min, at RT). The protein content was precipitated with acetone and 10% v/v trichloroacetic acid (16 h, at −20 °C). After centrifugation at 10,000*g* (at 4^o^C, 15 min), the supernatant was removed and the pellet rinsed with cold acetone. Alternatively, proteins were enriched using the Pierce Classic IP kit (replicates 5–7) before methanol precipitation. In all cases, the pellet was reconstituted in 50 μl of 80-mM ABC with 20% v/v acetonitrile and 3 μg of trypsin/LysC (Promega, V5071; high-specificity cleavage of C-term Arg and Lys residues with enhanced digestion efficiency at C-term Lys) were added for digestion (16 h, at 37 °C). Seven biological replicates in the WT, stress, and null were analyzed in triplicate by targeted LC-MS/MS on an Orbitrap Lumos mass spectrometer interfaced with the Easy-nLC 1200 system. Previously identified high-intensity peptides that are unique to OGT, HCF1, CARM1, histone-lysine N-methyltransferase SETD1A (SETD1A), GAPDH, histone deacetylase 1 (HDAC1), eukaryotic translation initiation factor 3 subunit B (eIF3b), and heat shock factor protein 1 (HSF1) were included for PRM analysis using a 0.4 m/z isolation window by higher energy collisional dissociation for 8 min surrounding the observed elution time (supplemental Table S4). Peptides were then separated over a 60-min gradient with a precursor ion scan from 350 to 2000 m/z, which was acquired at 120,000 resolution every 3 s followed by fragmentation scans acquired at 60,000 resolution. Data analysis was performed using Skyline (34, 35) and deposited in the PanoramaWeb portal (v20.7) with identifier https://panoramaweb.org/zyWhMm.url with ProteomeXchange accession PXD021091. The PRM assays fall under tier 3 of the molecular and cellular proteomics guidelines for targeted MS measurements of peptides and proteins.

Notably, we prepared samples using two different immunoprecipitation approaches. The data from replicates 5 to 7 are presented as these data demonstrated significantly higher peptide intensity than replicates 1 to 4; however, the data generated in replicates 1 to 4 mirror those in the latter replicates (supplemental Table S4).

### Experimental Design and Statistical Rationale

SILAC experiments were originally performed on four biological replicates with rotation of isotopic labeling across samples (see above). After LC-MS/MS analysis, total ion intensity, and correspondingly, protein identifications were significantly lower in one replicate indicating substantial sample loss (supplemental Table S1). Thus, this replicate was omitted from further analysis. Graphs and statistical analyses were prepared using GraphPad Prism software (v7). Protein interactor ratios are expressed as the median log2 fold change across three replicates.

PRM assays were performed on three biological replicates with at least 2 technical replicates per sample. Peak intensity was summed across peptides for each interactor. These samples did not make use of internal standards, nor were these samples isotopically labeled. Thus, to adjust inherent variabilities across immunoprecipitations from individual WT, H_2_O_2_, and null cell lysates, we chose to normalize each interactor by the summed peak intensities of OGT peptides (Fig. 7, *C–I*). Graphs were prepared using GraphPad Prism software (v7). Protein interactor validation is expressed as normalized peak intensity.

All remaining activity assays, immunoprecipitation, and Western blot experiments were performed in at least three biological replicates with controls as described above. Data are presented as the mean ± SEM unless otherwise indicated. Where relevant, a one-way ANOVA was applied (indicated in figure legend).

## Results

### OGT’s Activity Changes in Response to Stress

To begin to investigate the regulation of O-GlcNAc cycling in response to H_2_O_2_ treatment, we treated MEFs in which the OGT allele is flanked by lox recombination sites (20) with 2.5-mM H_2_O_2_ for 30 min to 2 h (22). Similar to prior studies, we observed a small decrease in protein O-GlcNAcylation followed by a modest, time-dependent increase in protein O-GlcNAcylation (Fig. 1*A*). Next, we measured OGT and OGA enzymatic activity using ^3^H-UDP-GlcNAc transfer to either CKII or α-crystallin peptides and the conversion of 4-MU-GlcNAc to 4-MU, respectively (Fig. 1*B*). Both OGT (CKII and α-crystallin) and OGA activities were moderately increased across all time points, but statistical significance was not reached (Fig. 1*B*). We note that the 120-min control exhibited modestly enhanced enzymatic activities of each enzyme, albeit to a lesser extent. This observation may indicate sensitivities of both enzymes to detect and respond to stress conditions that arise during cell treatment, such as mild temperature fluxes and changes in pH (1, 36, 37). Finally, we assessed the abundance of OGT, OGA, glutamine-fructose-6-phosphate transaminase (GFAT), and UDP-N-acetylhexosamine pyrophosphorylase (UAP1) (Fig. 1*C*). The latter two enzymes are components of the hexosamine biosynthetic pathway and thus regulate UDP-GlcNAc levels. These data did not identify changes in the abundance of OGT, OGA, GFAT, or UAP1. Collectively, these data suggest a more complex regulation of OGT and OGA during the cellular oxidative stress response.

### Identification of the Interaction Network of OGT

To investigate how OGT is regulated during oxidative stress, we used an SILAC-based MS approach to identify the protein interactors of OGT (Fig. 1*D*). MEFs were labeled with light, medium, and heavy isotopes of arginine (L: [^12^C_6_, ^14^N_4_], M: [^13^C_6_], H: [^13^C_6_, ^15^N_4_]) and lysine (L: [^12^C_6_, ^14^N_2_], M: [D_4_], H: [^13^C_6_, ^15^N_2_]). The inducible deletion of OGT (null) was used as a negative control. WT MEFs were treated with 2.5-mM H_2_O_2_ for 1.5 h, as this time point does not induce significant cell death but does show changes in O-GlcNAcylation (Fig. 1, *A* and *F*, and (22)). Cells were lysed and extracts from WT, H_2_O_2_-treated, and null cells were combined in equal amounts and precleared with isotype control rabbit antibody, followed by immunoprecipitation of endogenous OGT (Fig. 1, *D*, *E* and *F*). To further separate interactors, bound proteins were run on a large format 7.5% tris-glycine gel, stained with Coomassie brilliant blue and 16 similarly stained portions of the gel were excised (Fig. 1*E*, dash marks). Proteins were reduced, alkylated, and trypsin-digested in-gel followed by peptide extraction and contaminant removal by reversed-phase C_18_ chromatography. Peptides were further separated online by reversed-phase nano-LC and analyzed on an Orbitrap Lumos mass spectrometer. Additional characterization of the samples used for proteomic analysis demonstrated that the majority of OGT is enriched from SILAC lysates (Fig. 1*F*). Both the SYPRO Ruby protein stain and the Coomassie-stained gel indicate that OGT interacts with partners of varied molecular weights (Fig. 1, *E* and *F*), some of which are glycosylated (Fig. 1*F*).

Over 1800 proteins were identified with at least 1 unique peptide and 2 peptides total over 3 biological replicates (supplemental Table S1). For each replicate, the ratio of the WT or H_2_O_2_/null was log_2_ normalized and adjusted for the median to account for small variances when mixing isotopically labeled cell lysates before immunoprecipitation. Proteins found in ≥2 replicates with log_2_ WT or H_2_O_2_/null ≥1 were considered basal or stress-induced interactors, respectively.

**Fig. 2:**
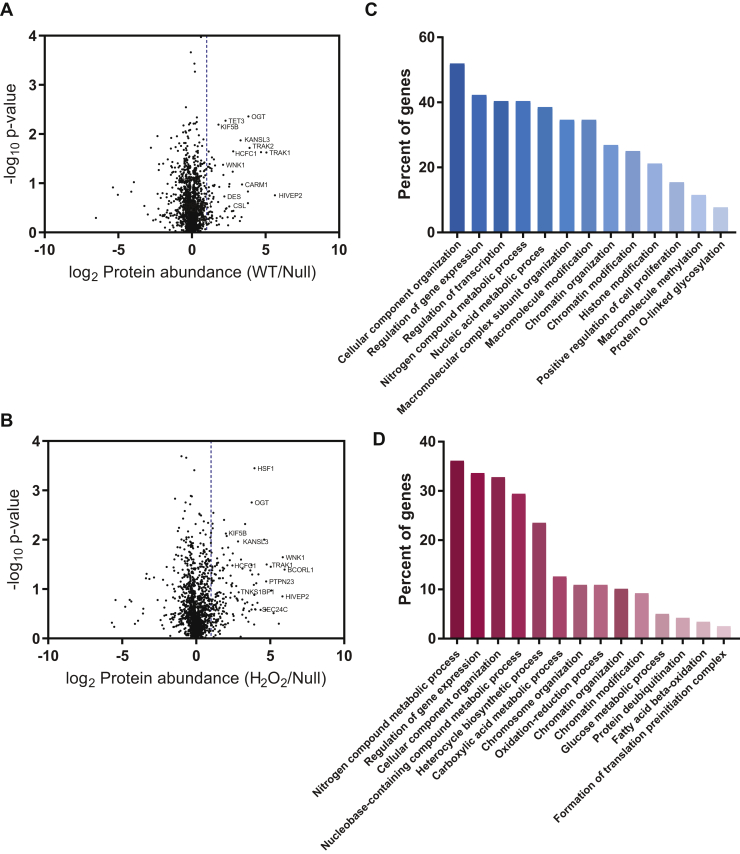
**OGT interacts with proteins with diverse functions basally and in response to stress.** Volcano plots of basal (*A*) and stress-induced interactions (*B*) for OGT. Median log_2_ protein abundance (WT/null, A; H_2_O_2_/null, *B*) for each interactor is plotted against –log_10_*p*-value across three replicates. The *dashed line* (x = 1) signifies threshold for true interactions. Gene ontology processes for basal (*C*) and stress-induced (*D*) interactions were analyzed by STRING enrichment with Cytoscape (*p* ≤ 0.05). The number of genes found in each process was normalized for the total input (percent of genes). H_2_O_2_, hydrogen peroxide; OGT, O-GlcNAc transferase.

Volcano plots of the -log_10_ *p*-value versus the median log_2_ WT/null or H_2_O_2_/null are plotted in Figure 2, *A* and *B*, respectively, where the dashed line indicates the threshold for basal or stress-induced interactions (x = 1). No *p*-value cutoff was applied, as several well-characterized interactors were below the typical threshold *p*-value of 0.05 such as CARM1 and desmin (18, 38). In total, OGT interacts with 47 proteins basally (Figs. 2*A* and 5C, supplemental Table S1) and 119 proteins upon oxidative stress (134 total, Figs. 2*B* and 5*C*, supplemental Table S1). An alternative experimental design would have been to enrich interacting proteins in parallel from WT, H_2_O_2_, and null samples and then to combine immunoprecipitates before analysis. This approach avoids interacting proteins in one isotopic label from associating with OGT from another isotopic label. However, this latter approach is subject to more technical errors. The consistency of our data and the number of proteins with a log_2_ WT or H_2_O_2_/null >1 suggest that a significant number of proteins originating from the null sample are not outcompeting interacting proteins from the WT and H_2_O_2_ samples. We note that the approach chosen is more likely to select for high-affinity interactors.

To assess the potential types of processes that are mediated by OGT via protein–protein interactions, the STRING functional enrichment application was used to identify gene ontology processes for the basal interactome (Fig. 2*C*). OGT interacts with diverse protein populations, a large percentage of which involve nucleic acid–mediated pathways, such as gene expression, transcription, chromatin and histone modifications, and chromatin organization. In addition, OGT interacts with proteins that regulate macromolecular complex formation. Similarly, stress-induced interactors also regulate gene expression and chromatin processes (Fig. 2*D*). However, stressed-derived interactors regulate novel processes including carboxylic acid metabolism (including the Krebs cycle), oxidation–reduction reactions, glucose metabolism, and fatty-acid beta oxidation (Fig. 2*D*).

**Fig. 3:**
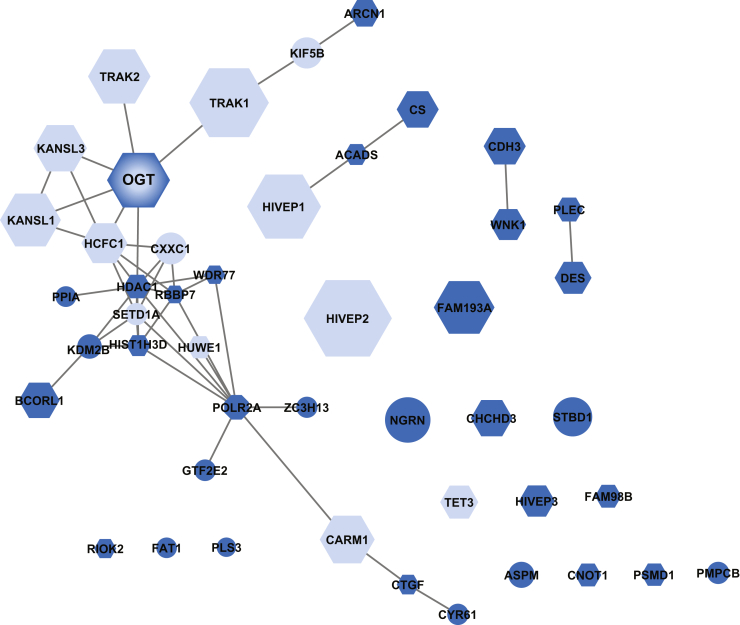
**OGT associates with various proteins at the basal cellular state.** Functional protein associations were analyzed using the STRING database in Cytoscape. The node size is indicative of WT/null ratio. Basal interactions were allocated into known (*light blue*) and novel (*blue*) categories, as well as assessed as targets for glycosylation by OGT (*hexagon*). OGT, O-GlcNAc transferase.

**Fig. 4:**
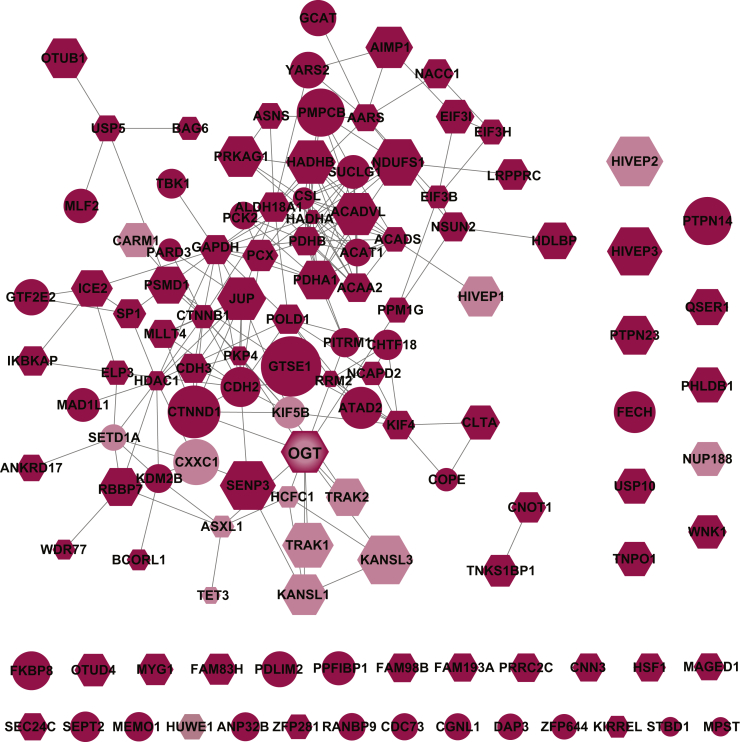
**OGT interacts with varied proteins in response to stress.** Functional protein associations were analyzed using the STRING database in Cytoscape. The node size is indicative of H_2_O_2_/null ratio. Stressed interactions were allocated into known (*pink*) and novel (*red*) categories, as well as assessed as targets for glycosylation by OGT (*hexagon*). H_2_O_2_, hydrogen peroxide; OGT, O-GlcNAc transferase.

The STRING enrichment application was used to generate networks based on OGT basal and stressed interactions within the Cytoscape molecular interaction platform. The node size is proportional to the log_2_ WT/null (Fig. 3) or H_2_O_2_/null (Fig. 4) ratios and thus may be an indicator of interactor abundance. Both basally and with stress, many known interactions such as KAT8 regulatory NSL complex subunits 1 and 3 (Figs. 3 and 4), as well as novel interactors such as G2 phase– and S phase–expressed protein 1 (Fig. 4), possess high log_2_ WT or H_2_O_2_/null (larger node size).

Several thousand glycosylation substrates have been defined for OGT. To determine if proteins in either the basal or stress-induced interactome were O-GlcNAcylated, we cross referenced our dataset with O-GlcNAcylation sites curated at PhosphoSite (39) or used manual literature curation. Of the 47 basal interactors, 30 (64%) have been previously characterized to contain O-GlcNAc, many with site-specific identification of the modified residue (Fig. 3, hexagon). A similar ratio was demonstrated with the stressed interactome, where 79 of the 119 stressed interactors (66%) have been demonstrated to be glycosylated (Fig. 4, hexagon). We also identified proteins that have been neither characterized as an OGT interactor nor as a substrate. These proteins include the protoporphyrin catalyzing enzyme, ferrochelatase, ATPase family AAA domain–containing protein 2, and tyrosine-protein phosphatase nonreceptor type 14.

Our interactomes were compared against the comprehensive protein interaction resource, Mentha (40), for OGT searched across *Homo sapiens, Mus musculus, Rattus rattus,* and *Caenorhabditis elegans*. Of the 158 previously published OGT interactions, 14 were identified in both our basal and stressed interactomes, with an additional two proteins, putative polycomb group protein ASXL1 and nucleoporin NUP188 homolog, unique to the stressed dataset (Fig. 3, light blue; Fig. 4, pink; supplemental Table S2). We also compared our interactomes with recent high-throughput screens for OGT interactions (18, 19, 41). This comparison identified HDAC1 in both interaction networks and ankyrin repeat domain–containing protein 17 and ran-binding protein 9 in only the stressed network (supplemental Table S2).

### Comparison of the Basal and Stress-Induced Interaction Networks

**Fig. 5:**
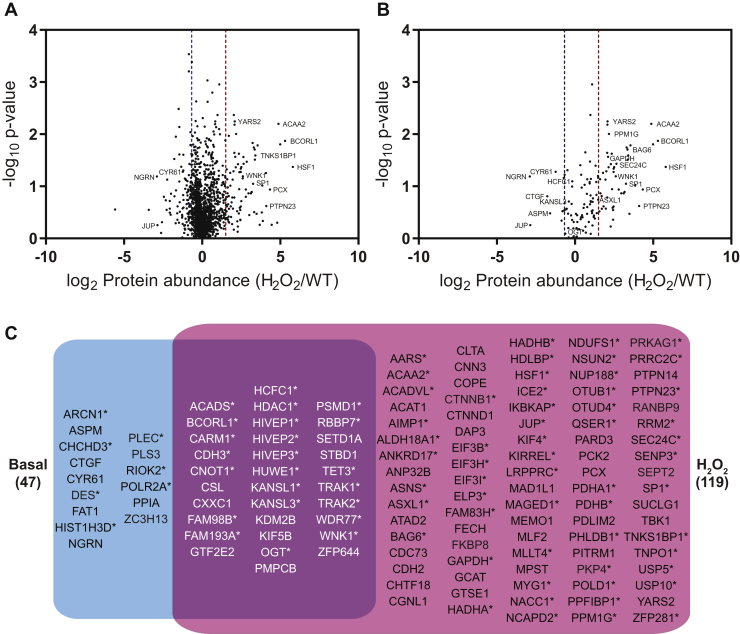
**The basal and stress-induced OGT interactomes contain shared and unique protein partners.***A* and *B*, volcano plots of OGT interactions in response to stress. Median log_2_ protein abundance (H_2_O_2_/WT) for each interactor is plotted against –log_10_*p*-value across 3 replicates. *A*, the plot represents all proteins identified by LC-MS/MS with one unique peptide and two peptides total. *B*, the plot represents interactors identified in at least 2 replicates where the log_2_ (WT or H_2_O_2_/null) ≥ 1. *Dashed bars* represent the threshold of stress-induced interactors (log_2_ WT/KO ≥ 1.5 or ≤ -0.67). *C*, basal and stress-induced interactions found in two or more replicates with a log_2_ (WT or H_2_O_2_/null) ≥ 1 were compared using Venny (v2.1) (https://bioinfogp.cnb.csic.es/tools/venny/). Proteins identified in both groups are highlighted in *white*. Glycosylated proteins are starred (∗). H_2_O_2_, hydrogen peroxide; OGT, O-GlcNAc transferase.

To evaluate changes in interactions with stress, we compared the log_2_ H_2_O_2_/WT for proteins from the basal and stress-induced interactomes for all proteins identified (Fig. 5*A*) and those that had been previously filtered for log_2_ WT or H_2_O_2_/null ≥1 in two or more (Fig. 5*B*). Proteins with median log_2_ H_2_O_2_/WT ≥ 1.5 were considered increased and those ≤ -0.67 were considered decreased (Fig. 5*B*; supplemental Table S1). In addition, proteins only found in the H_2_O_2_ treatment were considered increased, whereas those only identified in the WT group were considered decreased. In total, 92 proteins increased and 16 decreased their association with OGT upon stress.

Comparison of the 47 OGT basal interactors with the 119 interactors induced with H_2_O_2_ stress identified 32 shared proteins (Fig. 5*C*). The remaining 102 proteins, most of which are comprised of stress-induced interactors, are novel. These data are expected as no previous study has globally evaluated OGT’s interactions in response to oxidative stress. The novel stress-induced interactome includes proteins such as asparagine synthetase, leucine-rich PPR motif-containing protein, and phosphoenolpyruvate carboxykinase 2, which have varied responses within the cell during stress (42, 43, 44).

**Fig. 6:**
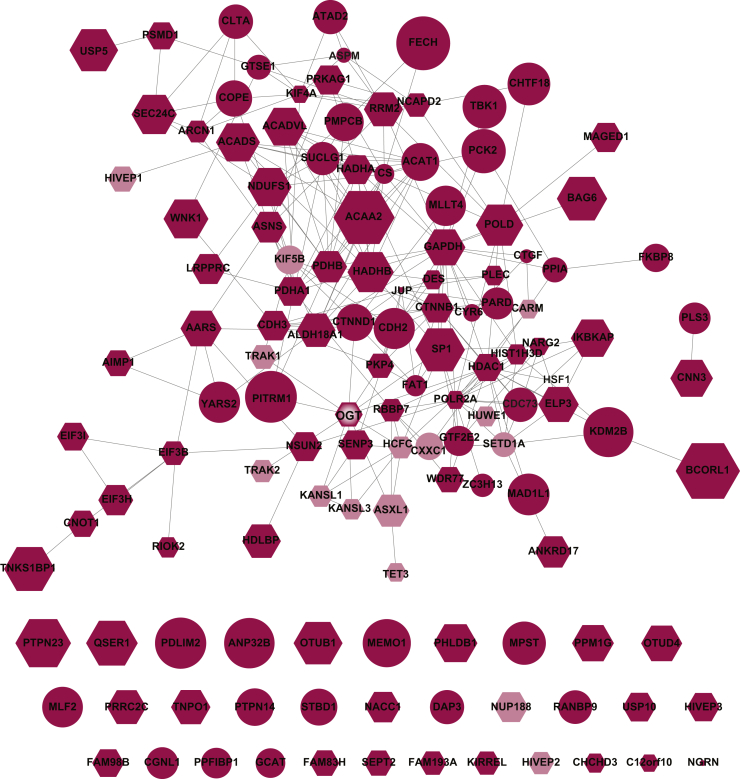
**OGT changes its interaction network in response to stress.** Functional protein associations were analyzed using the STRING database in Cytoscape for proteins with at least 2 replicates where the log_2_ (WT or H_2_O_2_/null) ≥ 1. The size of the nodes is indicative of the WT/H_2_O_2_ ratio. Stressed interactions were allocated into known (*pink*) and novel (*red*) categories, as well as assessed as targets for glycosylation by OGT (*hexagon*). H_2_O_2_, hydrogen peroxide; OGT, O-GlcNAc transferase.

All 134 basal and stress-induced interactors were analyzed with the STRING enrichment application (Fig. 6) where the node size is indicative of log_2_ H_2_O_2_/WT. Consistent with the Western blot analysis of OGT expression in response to stress over time (Fig. 1*C*), OGT levels as determined by LC-MS/MS indicate no difference between basal and stress states. As such, this node can be thought of ∼log_2_ H_2_O_2_/WT=0. Examination of the network reveals that most previously characterized interaction partners (pink), such as KAT8 regulatory NSL complex subunits 1 and 3, zinc finger protein 40, and kinesin heavy chain, are similarly sized to OGT, suggesting no difference in association in response to stress. In contrast, many novel interactors such as BCL-6 corepressor-like protein 1, TNKS1BP1, and 3-ketoacyl-CoA thiolase have high H_2_O_2_/WT ratios, specifying high OGT interactions with stress.

### OGT Interacts With Proteins Containing Various Structural Properties

As OGT interacts with proteins in varied cellular processes, but without apparent consensus sequence recognition, we sought to determine if specific domains, motifs, or functional regions (structural properties) were shared among interactors. OGT basal and stress-induced interactors were analyzed using PROSITE and UniProtKB bioinformatics tools (Table 2) (45, 46). We did not observe common structural properties among OGT interactors. Instead, OGT appears to interact with proteins containing diverse structural features that fall into broader categories based on functionality (supplemental Table S3), the largest of which are transcriptional and translational regulation, metal binding, and macromolecular complex formation/protein–protein interactions (Table 2). Nearly half (49%) of all interactors contain structural components that fall into the three aforementioned classifications, suggesting that these may be major functional categories for OGT-regulated processes. It is important to note that many proteins contain more than one domain or have unstructured regions. Thus, additional studies are required to define which protein regions comprise the protein–OGT interface.

**Table 2:** OGT interactors contain varied structural properties

Functional relevance	Gene name	Domains, motifs, and regions of similarity
Transcriptional, translational regulation	Aimp1, Ankrd17, Asxl1, Atad2, Carm1, Cxxc1, Gtf2e2, Hdlbp, Hist1h3d, Hivep1, Hivep2, Hivep3, Hsf1, Kdm2b, Lrpprc, Naac1, Nsun2, Pold1, Poldr2a, Ppfibp1, Rrm2, Setd1a, Sp1, Stbd1, Tet3, Wnk1, Zc3h13, Zfp281, Zfp644	Ben, bromodomain, BTB, DNA polymerase, histone H3, Hsf domain, JMJC, KH type, Lxxll motif, N4 Mtase, oxygenase domain, Phd, PRP, Ribored small, RNA Pol II repeat, SAM, SAM MT, Set, TFIIE Beta, Trbd, ZF C2H2, ZF C3H1, ZF CXXC, ZF PHD
Metal binding	Aars, Chd2, Chd3, Cnot1, Cxxc1, Eif3h, Fat1, Fech, Fkbp8, Hivep1, Hivep2, Hivep3, Kdm2b, Mpst, Ndufs1, Pcx, Pdlim2, Pls3, Pmpcb, Ppm1g, Rrm2, Setd1a, Sp1, Stbd1, Tet3, Usp5, Usp10, Yars, Zc3h13, Zpf281, Zfp644	FE2S Fer, 4FE4S HC3, 4FE4S MOW BIS MGD, AA tRNA Ligase, ATP grasp, cadherin, EF hand, ferrochelatase, FKBP PPIase, insulinase, lectin legume beta, Lim domain, Mpn, oxygenase, Post Set, PPM, rhodanese, Ribored small, Usp, ZF C2H2, ZF C3H1, ZF CXXC, ZF PHD, ZF UBP
Macromolecular complex formation; protein–protein interactions	Ankrd17, Anp32b, Arcn1, Asxl1, Bcorl1, Chchd3, Cope, Ctgf, Cyr61, Eif3h, Eif3i, Fat1, Fkbp8, Huwe1, Mllt4, Ogt, Pard3, Pdlim2, Plec, Ppfibp1, Ptpn23, Ranbp9, Rbbp7, Tnpo1, Usp5, Wdr77	Ank Rep region, B302 SPRY, Bro1, CHCH, importin B, Lam G domain, Lish, Lrr, Lxxll, MHD, MPN, PDZ, SAM, SH3, TPR, UBA, VWFC, WD repeats

OGT, O-GlcNAc transferase.

The specific domains, motifs, or functional regions of OGT interactors were analyzed with PROSITE and UniProtKB bioinformatics tools. OGT interactors contain many diverse structural properties, about half of which fall into three main categories: transcriptional/translational regulation, metal binding, and macromolecular complex formation/protein–protein interactions.

### Validation of OGT Interactors by Western Blot Analysis

**Fig. 7:**
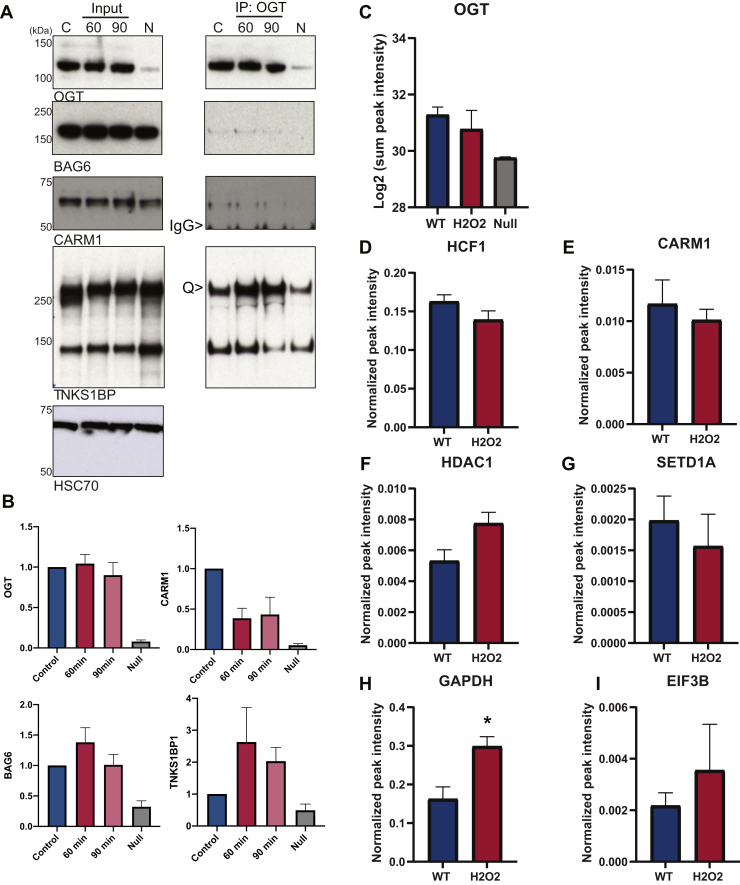
**OGT interacts with effectors in a stress-dependent manner.***A*, validation of OGT interactions with known effectors. OGT was immunoprecipitated (IP: OGT) from the untreated (C), H_2_O_2_-treated (2.5 mM, 60 min, and 90 min), and OGT null (N) MEF lysate (2 mg total) followed by Western blot for OGT, CARM1, TNKS1BP, and BAG6. Inputs (10 μg) were also probed for HSC70 (n = 3). IgG indicates the contaminating signal arising from the AL24 immunoglobulin used to enrich OGT complexes. *B*, the presence of OGT and its interactors in immunoprecipitates were quantified by densitometry and normalized to the WT control. Error bars represent the SEM. The antibody used to detect TNKS1BP detects two species; only the upper species which runs at the molecular weight of TNKS1BP was quantified (Q>). Inputs and IPs were taken from the same gel, but different exposures. OGT interacts with known and novel interactors as validated by PRM. OGT was immunoprecipitated independently from untreated (WT), H_2_O_2_-treated (2.5 mM, 1.5 h, H_2_O_2_), and null MEF cell lysates. After tryptic digestion, interactors were quantified using PRM: OGT (*C*), HCF1 (*D*), CARM1 (*E*), HDAC1 (*F*), SETD1A (*G*), GAPDH (*H*), and eIF3b (*I*). To normalize across immunoprecipitations, peptide intensities for each interactor were normalized to the intensities of two OGT peptides (AFLDSLPDVK and GSVAEAEDCYNTALR). Plotted WT (*blue*) and H_2_O_2_ (*red*), D-I. Error bars represent the SEM. ∗ *p* ≤ 0.05. Bag6, large proline-rich protein BAG6; CARM1, coactivator-associated arginine methyltransferase 1; eIF3b, eukaryotic translation initiation factor 3 subunit B; H_2_O_2_, hydrogen peroxide; HCF1, host cell factor 1; HDAC1, histone deacetylase 1; HSC70, heat shock cognate 71; IgG, immunoglobulin G; MEF, mouse embryonic fibroblast; OGT, O-GlcNAc transferase; PRM, parallel reaction monitoring; SETD1A, histone-lysine N-methyltransferase SETD1A; TNKS1BP1, 182-kDa tankyrase-1–binding protein.

**Fig. 8:**
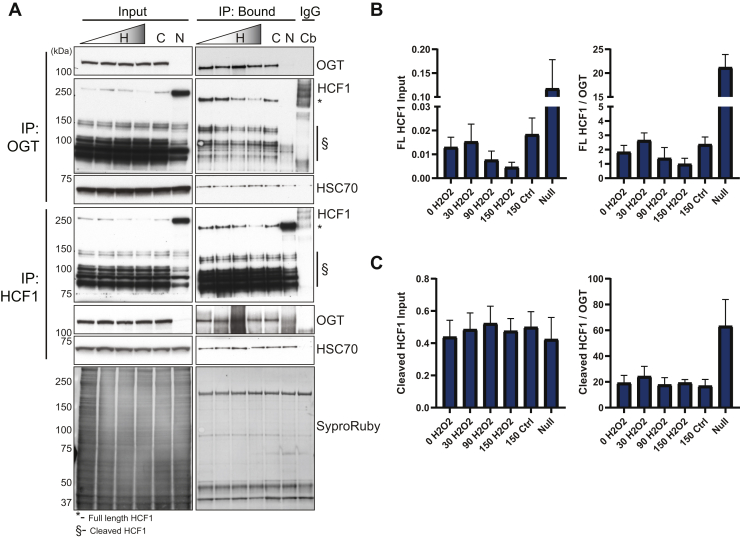
**HCF1 decreases its association with OGT upon stress.***A*, WT MEFs were treated with (H) or without (C) H_2_O_2_ (2.5 mM; lane 1, 0 min; lane 2, 30 min; lane 3, 90 min; lane 4, 150 min) and either OGT (*top panels*) or HCF1 (*bottom panels*) were enriched by immunoprecipitation from total cell lysates (300 μg) followed by Western blot for OGT, HCF1, and HSC70 (n = 3). As a control, lysate combined across all time points was immunoprecipitated (300 μg) with a nonspecific rabbit control isotype antibody (Cb). Input lysate is from 20 μg. HSC70 and SYPRO Ruby stained membrane function as loading controls. *B* and *C*, association of OGT remains constant with both FL and cleaved HCF1. From the HCF1 immunoprecipitation, FL and cleaved HCF1 were separately quantified in the input and bound fractions via densitometry. FL HCF1 (*B*) and cleaved HCF1 (*C*) were divided by the levels of OGT quantified in the bound fraction of the HCF1 immunoprecipitate (n = 3). HCF1 was normalized to SYPRO Ruby in the input fractions and to OGT in the immunoprecipitates. Error bars represent the SEM. FL, full length; H_2_O_2_, hydrogen peroxide; HCF1, host cell factor 1; HSC70, heat shock cognate 71; MEF, mouse embryonic fibroblast; OGT, O-GlcNAc transferase.

To validate known interactors, OGT was immunoprecipitated from WT, H_2_O_2_ (60 or 90 min), treated, and OGT null MEF lysate followed by Western blot analysis for interacting partners (Figs. 7 and 8). Both HCF1 and CARM1 are stable interacting partners and glycosubstrates of OGT (18, 19, 22, 47, 48, 49). In addition, OGT cleaves HCF1 along tandem repeats within HCF1, which remain associated with each other and are required for its transcriptional cofactor activity (50, 51). Consistent with the MS data, both CARM1 (Fig. 7, *A* and *B*) and HCF1 (Fig. 8) interact with OGT. TNKS1BP and BAG6 were also detected using co-immunoprecipitation (Fig. 7, *A* and *B*). These proteins demonstrate an increase in their association with OGT in response to oxidative stress (Fig. 7, *A* and *B*). Notably, all proteins demonstrate a strong fold enrichment in OGT WT cells when compared with OGT null cells (Fig. 7, *A* and *B*). We also note that TNKS1BP, BAG6, and CARM1 proteins demonstrate some variability in the timing of the stress-induced association or disassociation with OGT. We suspect that this variability stems from time-dependent dynamic interactions that are similar to the dynamics in O-GlcNAcylation in total cell lysates that we have reported previously (22). Here, O-GlcNAc levels are reduced transiently ∼60 min after the initiation of an oxidative stress; however, the timing of this reduction is variable. As H_2_O_2_ is inactivated at a rate proportional to the cell number, this variability likely arises from modest changes in the cell number stemming from technical errors or culture conditions. Taken together, these data recapitulate a subset of the data generated in the SILAC study.

### Validation of OGT Interactors by Characterization of the HCF1–OGT Interaction in Response to Stress

To further investigate the decreased association between HCF1 and OGT during stress, MEFs were treated with 2.5-mM H_2_O_2_ from 0 to 2.5 h. Both OGT and HCF1 were immunoprecipitated from the lysate derived from these cells followed by Western blot analysis (Fig. 8*A*). OGT immunoprecipitation (Fig. 8*A* top): OGT abundance (input) remains constant through all stress time points and results in comparable amounts of OGT immunoprecipitated regardless of time under stress (bound). Importantly, almost 100% of OGT is immunoprecipitated at all time points (unbound data not shown). FL HCF1 (Fig. 8*A*, HCF1 blot, 250 kDa, ∗) decreases linearly in response to stress, with corresponding decreases in HCF1 associated with OGT (bound). Conversely, the cleaved HCF1 products (Fig. 8*A*, HCF1 blot, 75–150 kDa, §) remain unchanged both in expression (input) and OGT association (bound). *HCF1* immunoprecipitation (Fig. 8*A* bottom): the same lysate was used for both OGT and HCF1 immunoprecipitations and thus the abundance (input) of both proteins is as described above. Immunoprecipitation of HCF1 resulted in close to full enrichment of the FL (250 kDa, ∗) and slightly less of the cleaved proteoforms (75–150 kDa, §), across all time points (unbound data not shown). FL HCF1, cleaved HCF1 proteoforms, and OGT were quantified in both the input and bound fractions (Fig. 8, *B* and *C*). Consistent with decreased FL HCF1 in response to stress (Fig. 8, *A* and *B*, input), the amount of FL HCF1 in the bound fraction correspondingly decreased. These data did not change when adjusted for OGT enriched after HCF1 immunoprecipitation (Fig. 8*B*). In contrast, the cleaved HCF1 proteoforms remain constant across stress time points, both in the input and bound fractions (Fig. 8, *A* and *C*). Together, these data suggest one of two possibilities: (1) HCF1 cleaved proteoforms comprise the majority of HCF1 protein, and thus, the decrease in FL HCF1 represents a very small percentage of what is associated with OGT or (2) a stable pool of OGT remains associated with FL HCF1, and when sufficient cleavage of HCF1 has been achieved, dissociation occurs.

### PRM

The propensity for a large proportion of OGT to be bound to HCF1 in the nucleus (52) coupled with the endogenous nature of our enrichment strategy limited our ability to confirm auxiliary interactors using a conventional immunoprecipitation–Western blot strategy. Thus, we used sensitive PRM to validate interacting partners in addition to HCF1 and CARM1, which included HSF1, HDAC1, SETD1A, GAPDH, and eIF3b (Fig. 7, *C-I*; replicates 5–7). Based on the SILAC data, unique peptides were chosen for each interactor and their m/z was monitored with high-resolution LC-MS/MS after OGT immunoprecipitation from independent WT, H_2_O_2_-treated, and null cell lysates. Interactors that had inconsistent peptide signal intensities were excluded from analysis (HSF1, ATPase regulatory subunit 13, data not shown). From SILAC and Western blot data, OGT levels do not change with stress (Fig. 1, C and F, 7, 8*A*) and were thus used as an internal control to normalize across immunoprecipitations (Fig. 7, C–I). As expected, lower peptide peak intensity was observed in the null for OGT (Fig. 7*C*), as well as interactors, and was thus not used to normalize immunoprecipitations in null cells. All interactors demonstrated significant fold change against the null, indicating that these proteins are OGT interactors rather than background (data not shown). Furthermore, most interactors show fold changes commensurate with the SILAC data (Fig. 7, *C–F*, *G*); the exceptions here is eIF3b (Fig. 7, *G* and *I*), which demonstrates no change with stress in the SILAC data compared with a modest increase after H_2_O_2_ stress in the PRM experiments (Fig. 7*I*). GAPDH demonstrates the most robust increase in association with OGT (Fig. 7*H*), consistent with SILAC data (supplemental Table S1).

## Discussion

These studies have defined the OGT interactome and the dynamic changes that occur upon oxidative stress, which is a key step to understanding how OGT is regulated to affect survival. Our previous studies, as well as those from other groups, suggest that OGT and OGA may be regulated locally by protein interactors (9, 11, 14, 16, 53, 54). Thus, we took a SILAC-based approach to identify both regulatory proteins that may mediate OGT’s function basally and proteins that are stress responsive and that may affect oxidative stress–induced injury. In total, we identified 134 protein interactors, many of which change their association upon stress, such as HCF1. These data delineate the endogenous basal interactome for OGT and determine which protein interactions are likely involved in the O-GlcNAc-mediated stress-induced survival response.

To understand the dynamics of cycling the O-GlcNAc modification during stress, we used a well-characterized model where subjecting cells to oxidative conditions induces a global stress response and increases O-GlcNAcylation (3, 22, 55). Our data are consistent with the studies by Lee et al that were completed with the same cellular system (MEFs), where increased O-GlcNAcylation is uncoupled from expression level changes in the enzymes that cycle the O-GlcNAc modification (22). Similar to the study by Groves et al, we observe a modest increase in OGT and OGA protein activity after H_2_O_2_ treatment, although not as significant as that observed in U2OS cells (16). One difference between these cell systems was that oxidative stress induced a modest change in the expression of OGT and OGA in U2OS cells. These differences in enzyme expression changes may be due in part to cell type–specific responses (MEF vs. U2OS). The latter is supported by observations in Cos-7 cells, in which H_2_O_2_ (1 mM, H_2_O_2_) treatment resulted in increased abundance of OGT protein (3), whereas treatment in SH-SY5Y (50 or 100 uM, H_2_O_2_), H_2_O_2_ resulted in a decrease in OGT expression (56).

As we cannot account for global changes in O-GlcNAc during stress because of either altered enzyme activity or abundance, we hypothesized that the enzymes that cycle the O-GlcNAc modification are regulated by protein interactions. To begin to address this, immunoprecipitation of OGT coupled with quantitative proteomics (SILAC) was used to identify over 1800 proteins over three biological replicates. A large number of false positives are to be expected because of the sensitivity of mass spectrometers and the nonspecific binding of resins and antibodies (57). Thus, we utilized MEFs where OGT can be inducibly deleted (OGT null) (20) as a control for nonspecific background. As complete deletion of OGT is lethal (20, 58), some OGT protein remains (Fig. 1*C*, *F*, 7, 8*A*) at the time point that the cells are harvested (∼40 h). As a consequence, some interactors still associate with the remaining OGT in the null cell, as can be seen with HCF1 and TNKS1BP (Figs. 7 and 8), likely resulting in some compression of the log2 fold change. Thus, after log_2_ normalization, we chose a twofold enrichment over the null as our threshold for positive interactions across two or more replicates. This minimum-fold increase allowed us to include well-characterized OGT interactors, such as trafficking kinesin-binding protein 1 and SETD1A, which had log_2_ WT/null=∼5 and 1, respectively. The range of ratios may be indicative of stoichiometry between OGT and a specific interactor, avidity in binding, or the rate of association and dissociation. From this analysis, we identified 47 basal interactors and 119 stress-induced interactors.

To our knowledge, this is the first analysis of endogenous OGT interactions in the cell, where changes in response to a stimulus, that is, oxidative stress, have been quantified in a comprehensive manner. The strategy used allowed us to assess *in vivo* interactions for OGT and circumvent disruptions in the cell cycle (59), circadian rhythm (60), and transcriptional processes (61) caused by overexpression of OGT. Nonetheless, previous work has contributed significantly to understanding OGT interactions in a variety of systems. Early studies by Cheung *et al*. (18) characterized 27 putative OGT-interacting proteins by yeast-two-hybrid (18, 62, 63), whereas more recent studies by Ruan (19) and Gao (41) utilized MS to identify global protein interactions of overexpressed, tagged OGT in HEK293T and HeLa cells, respectively. Of the 134 basal and stress-induced interactors, only 18 have been previously characterized to interact with OGT (13%). The lack of overlap between these datasets is likely due to different species and cell types, expression systems, and identification methodologies. Furthermore, most interactions previously mapped have been performed under basal conditions, whereas a majority of interactions mapped in this study arise under stress.

Analysis of the basal and stress-induced interaction networks revealed that OGT is proximal to previously characterized interactors, with novel interactors more distally located. These data are expected as STRING networks are generated based on literature curation, as well as predictive algorithms (32). Our data demonstrate that a high percentage of all interactors identified are also glycosylated (64% in total). Interestingly, glycosylation targets are spread throughout both networks, including to the most distal edges and unconnected nodes. These data indicate that OGT may interact sufficiently with substrates to withstand immunoprecipitation conditions and/or that substrates serve additional roles during their interactions with OGT. The percent of glycosylated substrates is similar between WT (30 total, 64%) and H_2_O_2_-treated (79 total, 66%) cellular lysate. Because there was a greater number of unique interactors with stress and, consequently, more total glycoproteins, it is possible that the increased global levels of glycosylation seen in most stress models are due to glycosylation of additional targets rather than an increased stoichiometry of glycosylation of a small pool of substrates. However, further studies with site-specific quantification are required to validate this hypothesis.

Gene ontology analysis using the STRING enrichment application revealed potential processes for OGT interactors that are conserved at a basal level through the induction of stress, including regulation of gene expression, cellular component organization, and chromatin organization and modification (Figs. 2*C* and 4). These data suggest that OGT mediates these essential, constitutive, regulatory processes. In contrast, during stress, the processes regulated by OGT switch to carboxylic acid metabolism, oxidation–reduction reactions, and regulating glucose and fatty acid metabolism. Collectively, these data support the hypothesis that there are many pools of the O-GlcNAc cycling enzymes bound in varied macromolecular complexes that can sense the cellular environment and rapidly respond to stimuli.

The N terminus of OGT (isoforms 1 and 3) contains 13 tetratricopeptide repeats (TPRs), which adopt a superhelical structure (14). Because of the initial cloning and characterization of OGT, it has been proposed that protein regulation of OGT occurs through TPR binding (9). Later studies demonstrated that specific TPR domains within OGT are essential for substrate recognition and that this requirement is relaxed with a reduced polypeptide size and structure (14). TPR domains do not recognize and bind proteins via the secondary structure but instead utilize distinct folds within the domain to generate binding pockets that allow for diverse protein interactions (64). PROSITE and UniProtKB analysis of the potential structural properties contained within OGT interactors was consistent with OGT binding to proteins with diverse structural properties, ranging from those involved in transcriptional/translational regulation, such as K homology and various zinc finger domains, to those involved in macromolecular complex formation, such as ankyrin and WD40 repeats (Table 2, supplemental Table S3). These data support the idea that the TPR domain is a scaffold for many protein interactions, allowing OGT to participate in diverse pathways within the cell. Additional studies are necessary to establish the specific OGT–protein interface for each interacting partner.

BAG6 (BAT3) and TNKS1BP both demonstrate enhanced interaction with OGT in response to injury, suggesting that these proteins regulate OGT, target OGT to substrates, or are substrates of OGT during injury (Fig. 7, *A* and *B*). BAG6 is a ubiquitin-like protein that is essential for protein quality control including synthesis of aggregation-prone polypeptides. Notably, OGT and O-GlcNAc have been demonstrated to play a role in mediating the stability of proteins with intrinsically disordered domains during translation (1). TNKS1BP is one component of the CCR4/NOT complex, components of which are targeted by OGT during oxidative stress (21). These data suggest that O-GlcNAc and OGT play a role in regulating mRNA metabolism during injury. Both HCF1 and CARM1 are characterized interactors and glycosylation targets for OGT (18, 19, 47, 48, 49). We identified and validated these interactors in our study, both of which showed decreased association with OGT after oxidative stress (Figs. 5, 7 and 8). Our data for CARM1 are consistent with similar studies by Lee et al, who observed reduced glycosylation of CARM1 in response to H_2_O_2_ stress (1 and 2 h) in the absence of alterations in CARM1 protein levels (22). These data may suggest that CARM1 dissociates from OGT with stress, diminishing its glycosylation. HCF1 is a transcriptional coregulator, requiring proteolytic maturation within 26 amino acid repeats. The proteolytic products (75–100 kDa) remain noncovalently associated (65, 66) and are required for HCF1 regulation of the cell cycle (67). In vertebrates, proteolysis of HCF1 is catalyzed by OGT (52) using the same active site that catalyzes glycosylation and requires UDP–GlcNAc as part of the catalytic mechanism (68). HCF is one of the most well-characterized interacting partners of OGT, with ∼50% of nuclear OGT stably associated with HCF1 (52). Our studies demonstrate a similar stoichiometry between the two proteins: OGT immunoprecipitation enriched for roughly 80 to 90% of FL HCF1 (data not shown), whereas cleaved HCF1 associates with OGT less. These data have several possible explanations: first, OGT has a preference in binding FL HCF1, where proteolysis results in the release of OGT; second, there is insufficient OGT in the cell to bind all HCF1 cleaved proteoforms at a given moment; and third, OGT and HCF1 may exist as multimers within the same complex, such that enrichment of OGT also enriches both FL and cleaved HCF1 proteoforms. Future studies utilizing proteolytically defective OGT (68), OGT overexpression, and stoichiometry analysis would help clarify these results. The reverse immunoprecipitation leads to a similar set of questions in trying to understand the stoichiometric and catalytic relationship between these two proteins. Despite a decrease in HCF1 abundance, OGT levels in cell lysates (input) and specifically associated with HCF1 (bound) remain unchanged with stress. These data are striking as we would normally anticipate a diminished abundance in the primary enriched protein to result in a corresponding decrease in associated proteins. The absence of this phenomenon suggests that either there is a stable pool of OGT associated with FL HCF1 that dissociates upon proteolysis or that FL HCF1 comprises only a minor fraction of all HCF1 and thus changes in OGT due to decreased HCF1 cannot be detected.

Our data demonstrated that HCF1 abundance in total cell lysates decreases after H_2_O_2_ stress (Fig. 8). These data cannot be attributed to OGT regulation of HCF1 directly or its transcription/translation, as the OGT null also displays decreased abundance in response to H_2_O_2_ stress (data not shown). HCF1 was initially described as a coregulator of immediate early genes during herpes simplex viral infection through the formation of a multiprotein complex with VP16 and Oct-1 (65). Since then, HCF1’s role as a transcriptional coactivator has grown to include regulation of both basal and stressed states. The latter work has occurred primarily in *C. elegans*, where mutations in hcf-1 that produce a null mutant extend life span by up to 40% and increase resistance to oxidative stress, suggesting that hcf-1 is a negative regulator of oxidant stress (69). Conversely, mutant ogt with decreased O-GlcNAcylation activity reduces life span and is hypersensitive to stress (70, 71). These two phenotypes appear to act in an opposing manner through the transcription factor daf-16 (FOXO in mammals), which mediates metabolism, immunity, and stress resistance genes, collectively regulating longevity in *C. elegans* (72, 73). This work has been partially recapitulated in mammalian systems, demonstrating direct interaction between FOXO3 and both FL and cleaved HCF-1 proteoforms, as well as glycosylation of Thr317 on FOXO in response to glucose, which in turn activates antioxidant responsive genes (74, 75). Together with the prior literature, our data may point toward a role for HCF1 as a negative regulator of stress by sequestering OGT from glycosylating other targets, such as FOXO. In the absence of HCF1, that is, dissociation between the two proteins induced by the stress response, OGT is now free to move about the nuclear and cytoplasmic regions of the cell, glycosylating stress responsive substrates to promote survival. Additional studies will be required to delineate the precise mechanisms by which HCF1 and OGT respond to stress to affect survival.

Owing to the robust relationship between OGT and HCF1, validation of additional interactors was challenging with traditional Western blot methodologies. Thus, we turned to label-free PRM to validate a subset of interactors, which has comparably higher specificity and sensitivity for protein quantitation than Western blotting (76). To our knowledge, this is the first study utilizing PRM to investigate OGT and its interaction networks. PRM allowed us to verify that OGT abundance is similar under basal and stressed conditions and that HCF1 and CARM1 decrease their association with OGT in response to an oxidative stress stimulus. In addition, we were able to validate that HDAC1 and GAPDH amplify OGT contact during stress, which is consistent with the SILAC study. In contrast, eIF3b, which remained unchanged in the SILAC study, showed elevated association with OGT after stress. The discrepancy in quantitation may be due to inherent differences between the methods, including increased spectral complexity with SILAC and the ‘missing value problem’ produced by the data-dependent analysis (77). In addition, differences between datasets may be due to the mixing of SILAC lysates before OGT enrichment, which likely enriches the most abundant and high-affinity interactors. Nonetheless, these data indicate that associations outside of the OGT–HCF1 paradigm may require the use of methodologies that are sufficiently sensitive to enable accurate characterization of the OGT–interactor relationship.

In summary, we have mapped the endogenous basal and stress-induced interactomes for OGT. Together, these data suggest that OGT exists in varied protein complexes within the cell that respond to stress stimuli. These studies will form the foundation for understanding how protein interactors impact OGT function *in vivo* to affect survival during oxidative stress–induced injury.

## Data Availability

SILAC experimental data have been deposited on ProteomeXchange Consortium via the PRIDE (30) partner repository with the dataset identifier PXD013645. PRM data have been deposited on the PanoramaWeb portal with the identifier https://panoramaweb.org/zyWhMm.url with corresponding ProteomeXchange accession PXD021091.

## Conflict of interest

The authors declare no competing interests.
